# Use of energy-dispersive X-ray fluorescence combined with chemometric modelling to classify honey according to botanical variety and geographical origin

**DOI:** 10.1007/s00216-019-02255-6

**Published:** 2019-11-25

**Authors:** Yiannis Fiamegos, Catalina Dumitrascu, Michele Ghidotti, Maria Beatriz de la Calle Guntiñas

**Affiliations:** European Commission, Joint Research Centre Geel, Retieseweg 111, 2440 Geel, Belgium

**Keywords:** Honey, Botanical variety, Geographical origin, Fraud, ED-XRF

## Abstract

Honey is one of the food commodities most frequently affected by fraud. Although addition of extraneous sugars is the most common type of fraud, analytical methods are also needed to detect origin masking and misdescription of botanical variety. In this work, multivariate analysis of the content of certain macro- and trace elements, determined by energy-dispersive X-ray fluorescence (ED-XRF) without any type of sample treatment, were used to classify honeys according to botanical variety and geographical origin. Principal component analysis (PCA) and partial least squares-discriminant analysis (PLS-DA) were used to create classification models for nine different botanical varieties—orange, robinia, lavender, rosemary, thyme, lime, chestnut, eucalyptus and manuka—and seven different geographical origins—Italy, Romania, Spain, Portugal, France, Hungary and New Zealand. Although characterised by 100% sensitivity, PCA models lacked specificity. The PLS-DA models constructed for specific combinations of botanical variety-country (BV-C) allowed the successful classification of honey samples, which was verified by external validation samples.

**Electronic supplementary material:**

The online version of this article (10.1007/s00216-019-02255-6) contains supplementary material, which is available to authorized users.

**Figure:**
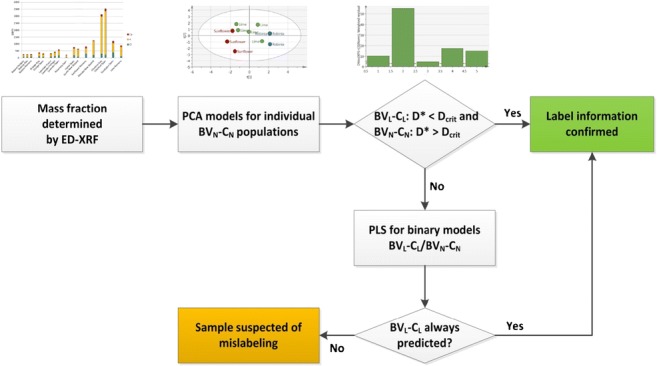
Graphical abstract

## Introduction

Honey is one of the food commodities more frequently affected by fraudulent activities [1]. This statement was confirmed by the outcome of a coordinated control plan on honey authenticity organised in 2015 by the European Commission. Around 20% of the analysed samples (blends of EU honeys, or originating from one specific Member State (MS)), very likely contained added sugars, such as corn syrups derived from starch and inverted sucrose syrup [2]. The outcome of a recent study carried out with Australian honeys [3] showed that about 27% of the commercially available honey samples analysed were of “questionable authenticity.”

In the European Union, Council Directive (2001/11/EC) [4] relating to honey, provides a definition of the product and the different accepted types, thresholds for physical and chemical parameters to be fulfilled by the different honey types, and kinds of processes that can be applied to honey without losing its classification as such. Any conflict between a honey sample and what is stipulated in Council Directive 2001/110/EC is considered a non-compliance.

Probably the most frequent type of adulteration in the honey sector is the addition of extraneous sugars that resemble the natural sugar composition of honey. From an analytical point of view, this type of adulteration can be detected by stable carbon isotope-ratio mass spectrometry (IRMS) [5], sometimes coupled to liquid chromatography [6, 7].

Council Directive 2001/110/EC requests information on the geographical origin of honey to be included in the labelling. False declarations of the geographical origin of honey and its botanical variety constitute another frequent type of adulteration. A thorough review of analytical methods for the determination of the geographical origin and botanical variety of honey has been published by Anklam [8]. Determining the elemental composition of honey is one of the more frequently used approaches for the characterisation of honey both in terms of its botanical variety [9] and its geographical origin [3, 10–12] and for discriminating honeydew from blossom honeys [13].

Inductively coupled plasma (ICP) coupled to atomic emission spectrometry (AES) [9, 11] or to mass spectrometry (MS) [12] are the analytical techniques most widely used for the determination of trace elements in honey. The use of other approaches such as total reflection X-ray fluorescence has also been described in the literature [10]. All the mentioned methods provide low limits of detection but require laborious sample pre-treatment. Those based on the use of ICP need sample digestion and the use of total reflection X-ray fluorescence requires the addition of an internal standard and, after equilibration, pre-concentration overnight.

The aim of the present work is to explore the possibility of using the elemental fingerprint of honey determined by energy-dispersive X-ray fluorescence (ED-XRF) to ascertain claims made on the label in terms of botanical variety and geographical origin. ED-XRF was used in the determination of macro-elements and trace elements in 70 honey samples of nine different botanical varieties (orange, robinia, lavender, rosemary, thyme, lime, eucalyptus, chestnut and manuka), originating from seven different geographical origins (Italy, Romania, Spain, Portugal, France, Hungary and New Zealand). Multivariate data analysis, i.e. principal component analysis (PCA) and partial least square-discriminant analysis (PLS-DA) was used to reduce the dimensionality of the data and for developing classification models.

## Materials and methods

### Honey samples

In 2015, in the frame of a coordinated control plan on honey authenticity, the JRC carried out a series of analysis whose main goal was to detect the presence of exogenous sugars in honey. The JRC had in this way access to several hundreds of honey samples commercialised in the Member States of the European Union.

In 2017, with the purpose of evaluating if the elemental composition could be used to classify them on the basis of botanical variety and/or geographical origin, some of the samples were also analysed by ED-XRF. Only mono-floral honey samples, from one single geographical origin (country) were selected for this study; poly-floral and mono-floral honeys in which honeys from different geographical origins had been blended, were excluded. Honey samples following the mentioned requirements purchased in Belgian and Spanish supermarkets in 2018 and 2019 were also included in the study (to construct and to validate the models), covering in this way likely variation in the honeys due to production year. Models for multivariate data analysis using PCA were constructed only if five or more honey samples belonging to a certain class (botanical variety and country of origin) were available; thus, the study was restricted to the following combinations botanical variety-country of origin (BV-C): orange-Italy (*n* = 6), orange-Spain (*n* = 9), lavender-France (*n* = 5), lavender-Portugal (*n* = 7), lavender-Spain (*n* = 5), rosemary-Spain (*n* = 5), robinia-Hungary (*n* = 11), robinia-Italy (*n* = 5), lime-Romania (*n* = 5), chestnut-Italy (*n* = 5) and manuka-New Zealand (*n* = 7). For PLS-DA modelling also groups for which only three samples were available, i.e. robinia-Romania, thyme-Spain, thyme-New Zealand, chestnut-Spain and eucalyptus-Spain were included in the study. The small number of samples available in some of the populations to construct the classification models could be a drawback. However, the way the samples have been acquired (some purchased in supermarkets in several countries and including different brands, some taken by inspectors in the frame of control plans) and the different production years covered could made them representative of the maximum variability to be expected in the different populations.

The botanical variety of a group of the honey samples taken in the frame of the 2015 coordinated control plan had been confirmed by pollen analysis (47 in total). For the remaining samples, the information given on the labels regarding botanical variety and for all of them on geographical origin was assumed to be correct.

### Reagents and standards

Blank measurements were run with deionised water from a Milli-Q Plus system (> 18.3 MΩ) (Millipore, Billerica, MA, USA).

A multielemental stock solution containing Ag, Al, B, Ba, Bi, Ca, Cd, Co, Cr, Cu, Fe, Ga, In, K, Li, Mg, Mn, Na, Ni, Pb, Sr, Tl and Zn at 1000 mg kg^−1^ each (Merck) and individual solutions of As, Br, Cl, Hg, Mo, P, Rb, S, Se, Sn, Ti and V 1000 mg kg^−1^ (Merck) were used to evaluate the bias introduced by the built-in Auto Quantify application of the ε5 software (PANalytical, Almelo, The Netherlands) used for quantification purposes.

### Instrumentation and sample preparation

An Epsilon 5 (PANalytical, Almelo, The Netherlands) ED-XRF instrument was used to carry out the analysis of honey. A detailed description of the instrument and the performance characteristics achieved in the analysis of solid samples is given elsewhere [14]. For the measurements of the honey samples, holders for liquid samples were used and the measurements were run in a helium atmosphere and not under vacuum as it is done for the analysis of solids. The bottom of the holders for liquid samples is a 6-μm polypropylene film that gave rise to blank values for P, Ca and Zn. To correct the contribution of the blank to the P, Ca and Zn results, deionised water was measured ten times and the mass fractions obtained were recorded.

Honey samples were kept at room temperature before analysis and aliquots were also taken at room temperature without warming up the honey. Approximately 15 g of honey were taken with a metal-free spatula and transferred into the liquid holders of the ED-XRF for measurement without undergoing any other type of pre-treatment.

To mimic the normal analytical process in a routine control laboratory, each sample was measured once and the mass fractions of trace elements in the honey samples were calculated using the calibration curves provided in the Auto Quantify Liquid application included in the ε5 software provided by PANalytical to run the Epsilon 5 instrument. Some experiments were carried out to evaluate the accuracy of the results measuring the commercial mono- and multielemental standard solutions of approximately 1000 μg kg^−1^ described in the “Reagents and standards” section, without further thorough validation. For multivariate analysis, the raw data obtained with the Auto Quantify application were used without any correction, avoiding in this way mathematical artefacts such as negative mass fractions due to the inherent standard deviation of the blank measurements. This could happen in honeys with low elemental mass fractions as it is the case for robinia honeys.

Quantifiable results in all or some of the honey samples were obtained for Al, P, Cl, K, Ca, Fe, Zn, Mn, Rb and Br. All of them were used in the multivariate analysis of the analytical data (construction of models) for classification purposes.

### Reproducibility evaluation

A thorough validation had been previously carried out for the analysis of solid samples using the ED-XRF instrument described above [14] which included the calculation of associated standard uncertainty. Within-pellet variation accounted for part of the standard uncertainty in solid sample analysis and since the measurement of the honey samples as liquids does not imply the preparation of pellets, the uncertainty of the Auto Quantify Liquid application used in this work could be different.

To be sure that the difference in the elemental composition of different honeys is due to true variations between honeys, and not to the result of poor analytical precision, reproducibility studies were carried out. Four different honeys—robinia, orange, lime and chestnut—were measured in triplicate in three different days. Precision studies were carried out on the data as directly obtained from the Auto Quantify Liquid application without correction for bias and/or blank because those were the data used in the creation of the models as said above.

Aliquots of approximately 15 g of each one of the four honey samples were transferred to liquid holders in triplicate and each holder was measured only once, reproducing in this way the approach described in the “Instrumentation and sample preparation” section. Accordingly, the total amount of samples measured each day was 12. The measurements were repeated on three different days. At the end of the study nine results per element and per honey type were available. ANOVA (95% confidence interval, CI) was used to calculate the reproducibility values summarised in Table 1, which were in good agreement with those published earlier for solid samples [14], keeping in mind the low concentrations of some elements in honey. The reproducibility values within elements were quite similar among the different types of honey, the largest difference being obtained for Cl in lime honey; this could be due to some dark particles in suspension that were observed in this honey and that could increase the heterogeneity in the sample.

**Table 1:** Reproducibility values in % for the different elements analysed in robinia, orange, lime and chestnut honey

	P^a^		Cl		K		Ca^a^		Cu		Zn^a^		Fe		Rb		Mn	
	ppm	Rep. (%)	ppm	Rep. (%)	ppm	Rep. (%)	ppm	Rep. (%)	ppm	Rep. (%)	ppm	Rep. (%)	ppm	Rep. (%)	ppm	Rep. (%)	ppm	Rep. (%)
Robinia	362.34	4.1	17.55	8.6	160.56	2	206.35	1.9	3.11	13.8	1.89	7.2	n.d.		n.d.		n.d.	
Orange	351.64	3.3	15.07	12	134.25	1.6	218.15	2.5	3.09	12.2	1.92	7.8	0.89	11.5	n.d.		n.d.	
Lime	364.65	5	95.64	19	663.55	1.1	245.53	2.9	3.04	13.9	2.08	12.5	3.55	12.9	n.d.		n.d.	
Chestnut	377.42	4.2	112.71	4	1074.61	0.5	247.73	3.3	3.84	13.6	2.33	6.5	1.29	12.8	3.61	2.7	4.40	4.6

Elemental concentrations as obtained from the Auto Quantify Liquid application, without correction for bias

*Rep.* reproducibility

^a^P, Ca and Zn concentrations are not corrected for the blank contributions and so are not those in the honey sample but include the contribution of the 6 μm polypropylene film

*t* tests (95% CI) were carried out using the results obtained during the precision study to evaluate if significantly different results for the elements analysed were obtained for the four honey samples: acacia, orange, lime and chestnut. Cl, K and Fe were significantly different for the four samples. For Fe, the *t* test was only run for orange, lime and chestnut because Fe was not detected in the acacia honey. Chestnut honey was significantly different from the other honeys for all elements, with the exception of Ca for which similar contents were found in chestnut and lime honey. *t* tests were not run for Mn and Rb because they were only detected in the chestnut honey. These preliminary results supported the idea that elemental composition determined by ED-XRF could be used for the purpose of honey classification. *t* tests were not carried out for all the possible BV-C combinations, which would have been a tedious, time consuming procedure, and multivariate analysis as described hereafter was used for classification purposes.

### Multivariate analysis

Multivariate analysis was carried out using the software SIMCA Version 15.0.2, Umetrics (Malmö, Sweden). Principal component analysis (PCA) was used to build up models for the different groups of honey defined by BV-C [15]. PCA models were used to identify outliers in the different populations making use of the Mahalanobis distance (DModX) (distance between a point and a distribution) (https://www.itl.nist.gov/div898/software/dataplot/refman2/auxillar/matrdist.htm) with a 95% significance level and to reduce the number of variables (elements) used to construct the models by means of loading plots. Elements situated in the loading plot in the centre of the coordinates or very close to them were removed, and the model was built up again in an iterative process. On the basis of DModX, none of the samples used for the construction of the models was flagged as outlier and all of them were kept for further studies.

To avoid overfitting, taking into consideration the rather limited number of honey samples used to construct the different models, the number of principal components used was kept to a maximum of three in most cases, and only in three cases (robinia-Hungary, orange-Spain and rosemary-Spain) four principal components were used.

For some but not for all BV-C combinations, PCA models alone allowed the successful identification of honey samples that did not belong to the population for which the model was constructed. PLS-DA was used to improve the rate of successful classifications. The variables used to construct the PLS-DA models were optimised using the loading plots. The maximum amount of components used in PLS-DA was three, and in some cases, one component was enough for prediction purposes.

The small number of honey samples in most of the models limited the statistical significance of cross-validation as carried out by the SIMCA software [16]. For this reason, external validation was carried out with a restricted number of samples bought in January–February 2019 in Belgium covering ten different BV-C combinations: orange-Spain (*n* = 2), lavender-Spain (*n* = 1), rosemary-Spain (*n* = 1), thyme-Spain (*n* = 1), chestnut-Spain (*n* = 1), eucalyptus-Spain (*n* = 1), eucalyptus-Italy (*n* = 1), lime-Romania, robinia-Romania and robinia-Italy (*n* = 1). Unfortunately, it was not possible to find appropriate samples to carry out the external validation of all the constructed classification models.

## Results and discussion

### Determination of trace elements by ED-XRF

It is not the purpose of this work to do a thorough characterisation of the macro- and trace element content of different honeys on the basis of their botanical variety, geographical origin or a combination of both but rather to demonstrate that the elemental fingerprint of honey can be used by routine control laboratories to classify honeys in the frame of anti-fraud activities. For this reason, the built-in Auto Quantify Liquid application of ε5 was used for quantification purposes without any further validation than what is described in the “Honey samples” and “Instrumentation and sample preparation” sections. The Auto Quantify Liquid application has been optimised by the manufacturer of the instrument for the analysis of industrial oils and fuels, matrices commonly analysed by ED-XRF and could therefore introduce significant bias when applied to other type of matrices, such as honey. Indeed, the recovery on standard aqueous solutions was on average 50 ± 10%, depending on the element. Table 2 shows the mass fraction ranges obtained for the different elements in the different honey groups after subtraction of the blank mass fractions for Ca and Zn and application of the correction factors derived from the analysis of the standard solutions. Since no thorough validation was carried out for the analysis of liquid samples, the results given in Table 2 can only be considered as indicative. ED-XRF does not allow the accurate determination of light elements. The first element that can be analysed with the instrument used in this study is Mg. However, Mg determination is characterised by high limits of quantification [14] and was only detected in one Romanian robinia honey. Al and P were the first elements in the periodic table that were quantified in a number of honey samples. In the case of P, not all the honeys provided results clearly distinct from the blank values introduced by the polypropylene film used to hold the liquid samples. Contrary to P that played an important role in the discriminative power of most of the models, Al was frequently situated in the centre of the coordinates of the loading plot and was hence eliminated from most of the models. Although the mass fractions obtained for Al and P were used for multivariate analysis (construction of models), they are not included in Table 2 because a thorough validation of the quantification method and characterisation of the blank would be needed to obtain accurate results about the concentrations that are characteristic of the different types of honey included in this study.

**Table 2:** Ranges of mass fractions of elements found in the different honey groups defined by botanical variety and country

	Cl	K	Ca	Fe	Zn	Mn	Rb
Robinia-Hungary (*n* = 11)	51.2–357.2	132.4–313.9	19.7–126.9	1.5–5.5	0.3–2.4		
Robinia-Italy (*n* = 5)	36.1–96.0	137.0–380.6	4.7–17.2	1.4–2.0	0.6–1.3		
Robinia-Romania (*n* = 3)	65.4–67.6	143.9–160.3	20.9–37.6	2.1–2.3	0.6–1.1		
Orange-Italy (*n* = 6)	50.0–286.3	137.3–368.2	25.9–59.9	1.7–3.4	0.4–1.4	1.8^a^	
Orange-Spain (*n* = 9)	49.5–68.7	180.0–271.6	39.2–65.5	1.9–3.5	0.4–1.6		
Lavender-France (*n* = 5)	62.6–113.9	167.5–287.9	29.9–46.6	1.7–3.3	0.6–0.9	1.0 ^a^	
Lavender-Portugal (*n* = 6)	93.6–117.0	202.6–316.4	19.5–47.8	1.7–2.2	0.5–2.5	1.6–2.3 (*n* = 5)^b^	1.8^a^
Lavender-Spain (*n* = 5)	88.9–362.5	295.7–1243.1	20.6–81.3	2.3–6.3	0.8–3.4	1.1–6.2 (*n* = 3)^b^	
Rosemary-Spain (*n* = 7)	34.3–119.2	86.4–183.8	17.0–83.7	1.4–2.9	0.2–1.0		
Thyme-Spain (*n* = 3)	128.8–284.0	435.6–599.0	62.3–111.5	3.1–3.7	1.1–1.7	1.9^a^	
Thyme-New Zealand (*n* = 3)	48.1–100.8	450.0–553.8	21.0–62.9	1.9–3.4	0.1–0.7	1.1–2.5 (*n* = 2)^b^	
Manuka-New Zealand (*n* = 7)	180.1–481.6	446.5–1640.4	31.5–59.0	1.5–3.5	1.0–2.0	1.6–12.8 (*n* = 6)^b^	4.0–6.0 (*n* = 5) ^b^
Chestnut-Italy (*n* = 5)	172.4–575.7	1883.8–3324.1	101.0–182.8	2.2–3.6	0.8–2.2	1.9–16.7	8.6–22.0
Chestnut-Spain (*n* = 3)	147.1–273.5	1730.5–3451.3	92.3–187.7	2.9–5.8	0.8–2.4	4.3–28.4	7.2–16.14
Eucalyptus-Spain *(n* = 3)	326.7–443.6	408.1–740.5	92.9–122.9	3.0–5.8	1.13–4.3	3.8–5.8 (*n* = 2)^b^	1.8–6.6 (*n* = 2)^b^
Lime-Romania (*n* = 5)	86.5–217.7	193.0–1034.9	41.1–110.7	1.7–10.3	0.3–1.3		
Sunflower-Romania (*n* = 3)	208.6–360.6	293.0–360.6	118.5–127.2	2.2–3.7	1.3–2.6	2.1^a^	1.6^a^

^a^Mass fraction found only in one honey in the full group. The mass fractions for that element in the rest of the honeys was < LoD (around 0.1 mg kg^−1^)

^b^Value between brackets: number of honeys in that population in which a certain element was quantified, if different from the total amount of samples in the population

**Fig. 1:**
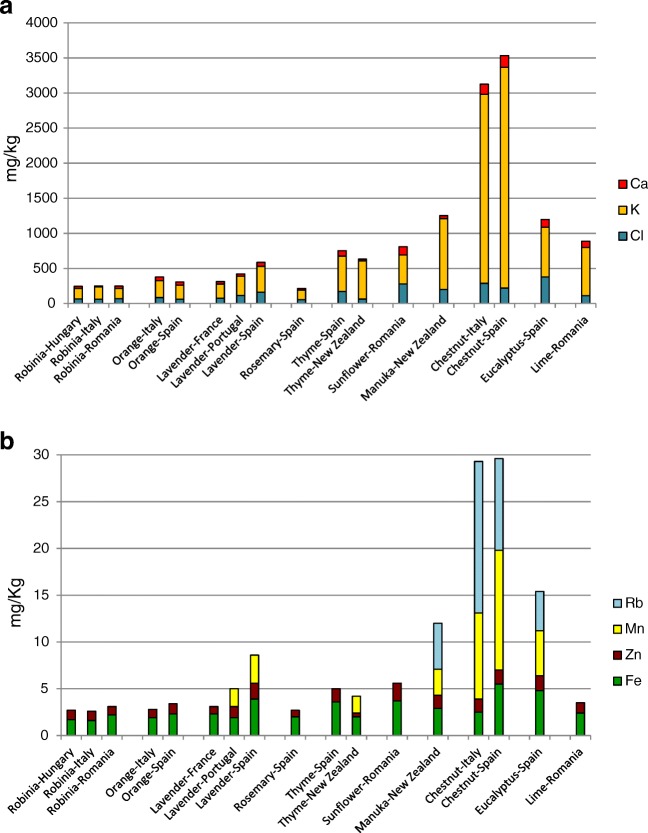
Medians of element mass fractions of the honey samples used in the construction of models: **a** major elements, **b** trace elements

Figure 1 shows the medians obtained for the Cl, K, Ca, Fe, Zn, Mn and Rb mass fractions in the different BV-C populations. In such small populations as those in this study, the mean mass fractions would be strongly affected by the presence of one sample with extremely high or low values. For this reason, Fig. 1 was constructed using the median as a robust estimator of location. Figure 1 shows some clear tendencies in the mass fractions of the different honeys, certainly on the basis of their botanical variety. As expected [9–11, 13], dark honeys such as chestnut, eucalyptus, lime and manuka have higher mass fractions than the typical light honeys (robinia, orange, lavender and rosemary) for all of the quantified elements. Chestnut honeys have by far the highest total content of macro- and trace elements, being particularly rich in K, the most abundant element in all the honeys analysed. On the other extreme of the total mass fraction range, robinia and rosemary honeys were those with the lowest values. Interestingly, thyme and sunflower honeys are light honeys with a total content of the quantified elements comparable to lime honeys which, among the dark ones, are those with the lowest total elemental mass fraction.

All the thyme honeys analysed have a distinct, characteristic elemental composition; the six samples analysed were very similar in terms of total elemental content and individual macro-elemental composition, even if originating from far apart countries, Spain (*n* = 3) and New Zealand (*n* = 3). This shows once again that the botanical variety has a stronger impact on the elemental composition of honey than the geographical region where the honey was produced. It is also interesting to observe the similar total, macro- and trace elemental composition of manuka and eucalyptus, two species which are autochthonous to Oceania.

The largest variation in element content was found for K, while it was lower for Cl, Zn and Fe.

The observed differences in elemental mass fractions were mainly caused by botanical variety and to a lesser extent by geographical origin. Nevertheless, some tendencies can be observed: for instance, the lavender honeys from the Iberian Peninsula were richer in macro- and trace elements than those from France, and the Spanish lavender honeys had higher mass fractions for all the tested elements than those from Portugal. Generally, the mass fraction of Cl was lower in Spanish orange and chestnut honeys than in the equivalent honeys from Italy. The same effect was observed for eucalyptus honeys (data not shown) which indicates that in general Spanish honeys have a lower Cl content than the respective Italian ones.

Nevertheless, the differences in macro- and trace element mass fractions among the various populations were not large enough to be used for classification purposes and for this reason, multivariate data analysis in the form of PCA (non-supervised algorithm) and PLS-DA (supervised algorithm) was carried out.

### Multivariate analysis of data

#### Principal component analysis

**Fig. 2:**
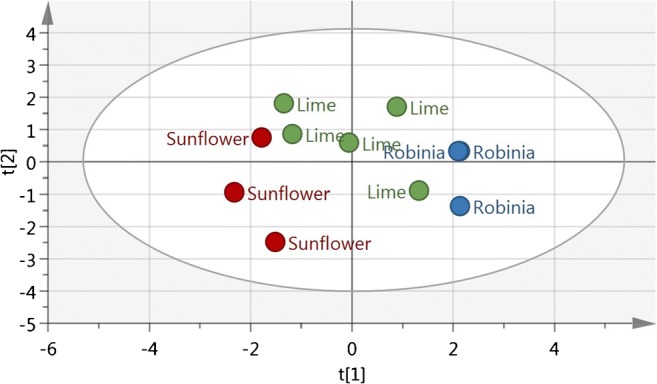
Score plot of three different monovarietal Romanian honeys: 3 sunflower, 5 lime and 3 robinia. t[1] and t[2] refer to the 1st and 2nd principal components respectively

The elemental composition of honey has been previously used to classify honey according to both geographical origin and botanical composition. In the present study, PCA models were constructed for honeys belonging to a certain botanical variety irrespective of their geographical origins (countries). The degree of model fit was, however, poor (low explained variation, *R*^2^*X*) as was the predictive ability of the model (low predicted variation, *Q*^2^*X*). In a second attempt, honeys were classified according to their geographical origin (country), each group combining honeys of different botanical variety. The results obtained were even less satisfactory than those for the classification according to botanical variety. However, in the PCA models for countries from which different botanical varieties were available, for instance Romania (robinia, sunflower and lime), tendencies to cluster according to botanical variety were observed (Fig. 2).

For that reason, models were constructed for honey groups defined by specific BV-C combinations. PCA models were constructed only for those groups in which at least five observations (honeys) were available, those were as follows: robinia-Hungary (*n* = 11), robinia-Italy (*n* = 5), orange-Italy (*n* = 6), chestnut-Italy (*n* = 5), lavender-France (*n* = 5), lavender-Portugal (*n* = 6), lavender-Spain (*n* = 5), orange-Spain (*n* = 9), rosemary-Spain (*n* = 7), lime-Romania (*n* = 5) and manuka-New Zealand (*n* = 7). Information about the mentioned PCA models is given in Table 3. Other combinations available with less than 5 honeys per group—chestnut-Spain (*n* = 3), eucalyptus-Spain (*n* = 3), thyme-Spain (*n* = 3), thyme-New Zealand (*n* = 3), sunflower-Romania (*n* = 3) and robinia-Romania (*n* = 3)—were used for external validation purposes to test false negatives and to construct PLS-DA models.

**Table 3:** Parameters defining the PCA models for the different honey populations studied.

	*R*^2^*X* [1]	*R*^2^*X* [2]	*R*^2^*X* [3]	*R*^2^*X* [4]	*Q*^2^*X* (cum)
Robinia-Hungary	0.559	0.204	0.126	0.0899	0.534
Robinia-Italy	0.643	0.217	0.121		0.577
Orange-Italy	0.425	0.303	0.154		− 0.331
Orange-Spain	0.486	0.284	0.107	0.0811	0.280
Lavender-France	0.658	0.284			0.690
Lavender-Portugal	0.624	0.260	0.0777		0.605
Lavender-Spain	0.667	0.227	0.0894		0.632
Rosemary-Spain	0.391	0.315	0.179	0.0939	0.454
Chestnut-Italy	0.589	0.257	0.114		0.450
Lime-Romania	0.631	0.296	0.0609		0.660
Manuka-New Zealand	0.458	0.262	0.156		− 0.159

*R*^*2*^*X* explained variation, *Q*^*2*^*X* predicted variation

The predictive ability of the models for orange-Italy and manuka-New Zealand was poor and did not improve by increasing the number of principal components. No clear explanation on the basis of the elemental mass fractions was found for the poor predictive ability of the manuka-New Zealand model. One possible general explanation could be the different regions within a country where the honeys could have been produced. This hypothesis would also explain the poor predictive ability of some models for Spanish honeys, namely, orange and rosemary, which can be produced in different regions in Spain. Unfortunately, specific information about the production region was only available for a limited number of samples. The botanical variety as such may also play a role.

It needs to be kept in mind that for most of the samples analysed, the information provided on the labels on botanical variety and geographical origin was considered to be correct. Only two out of the six honeys used to construct the orange-Italy model and three out of the seven included in the manuka model had been characterised by pollen analysis. If some of the honeys were misdescribed, they would have been wrongly included in a certain population affecting the quality of the model and its prediction capacity.

An accurate classification model is defined by high sensitivity and specificity, sensitivity being the rate of samples correctly assigned to a certain population (= true positives) and specificity the rate of samples correctly identified as not belonging to a population (= true negatives).

The accuracy of the PCA models created for the different BV-C groups was evaluated by means of the distance of a sample to the model DModX PS+ (95 % confidence interval): every sample with a DModX PS+ > *D*_crit_ does not belong to that population, while samples with a DModX PS+ < *D*_crit_ belong to that population.

The sensitivity of the PCA models was tested analysing honey samples that were purchased in Belgium at the beginning of 2019 and that were commercialised under brand names not used in the construction of the models. Assuming that the information provided on the labels was correct, the samples tested were as follows: orange-Spain (*n* = 2), lavender-Spain (*n* = 1), rosemary-Spain (*n* = 1), thyme-Spain (*n* = 1), chestnut-Spain (*n* = 1), eucalyptus-Spain (*n* = 1), eucalyptus-Italy (*n* = 1), robinia-Italy (*n* = 1), robinia-Romania (*n* = 1) and lime-Romania (*n* = 1). The sensitivity of the PCA models was 100% because all honeys were correctly classified by using the respective BV-C models.

Regarding specificity, the results were not always equally satisfactory. Some models were 100% specific; one example is shown in Fig. S1 in the Electronic Supplementary Material (ESM) in which all Spanish lavender honeys were clearly considered outliers by the orange-Spain and the lavender-Portugal honeys models. The high specificity of the lavender-Portugal model was confirmed with further tests (data not shown), since all the lavender-France honeys were also considered outliers by the lavender-Portugal PCA model. The orange-Spain model was also highly specific because all other Spanish honeys (chestnut, eucalyptus, thyme and rosemary) were flagged as outliers with the exception of two rosemary honeys, which corresponds to a specificity of 90%. Also all orange-Italy honeys were flagged as outliers by the orange-Spain model.

**Fig. 3:**
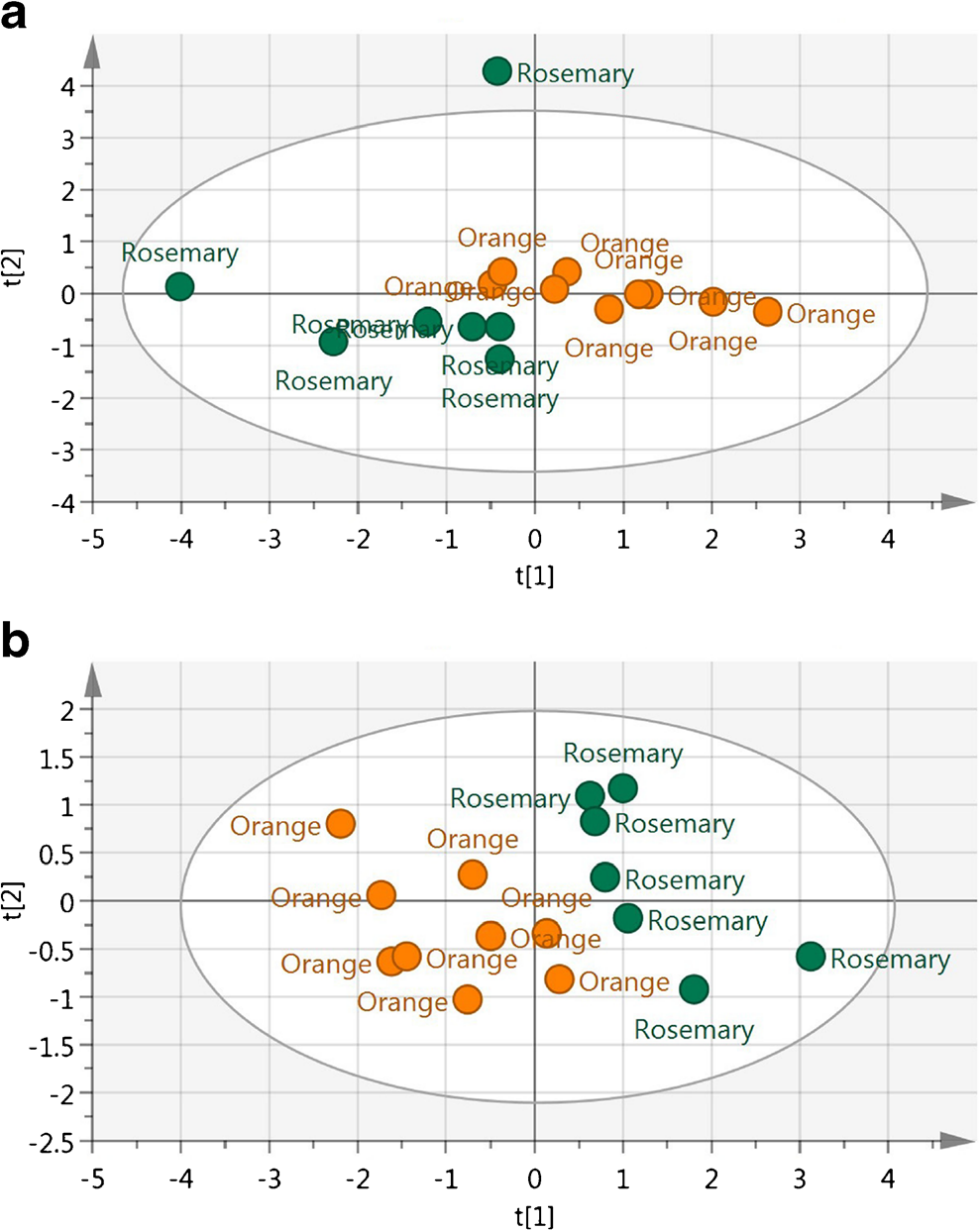
**a** PCA and **b** PLS plots of orange-Spain and rosemary-Spain honeys. t[1] and t[2] refer to the 1st and 2nd principal components respectively

Unfortunately, not all models were equally specific; for instance, the lavender-France model did not flag one lavender-Spain and two lavender-Portugal honeys as outliers, which correspond to a specificity of 75%, and the rosemary-Spain model did not consider any of the orange-Spain honeys as outliers. Figure 3a shows that PCA did not allow the full resolution of the two populations and that a certain overlap occurred. Given the poor prediction capability of the orange-Italy model, all orange-Spain honeys were accepted as being part of the orange-Italy population. In summary, several models showed 100% specificity towards some BV-C combination but frequently not towards all of them. It was then concluded that PCA models lacked the required specificity to scrutinise the correctness of label declarations regarding botanical variety and geographical origin. The same conclusion has been previously reached by Krops et al. [10] when classifying robinia, lime and chestnut honeys from different Slovenian locations, who finally used linear discriminant analysis (LDA); approach also followed in other works [9, 11]. Also Zhou et al. [3] concluded that no visual clustering of honeys of different geographical origin was achieved by PCA, even using six principal components, making use in this case of canonical discriminant analysis (CDA) for classification purposes. Support vector machine, multilayer perceptron and random forest were used by Batista et al. [12].

#### Partial least square-discriminant analysis

To improve the classification performance, supervised PLS-DA models were created to maximise the variance between classes. Figure 3 provides an example of the increased discrimination power achieved using PLS-DA instead of PCA when applied to the populations of orange-Spain and rosemary-Spain honeys.

**Fig. 4:**
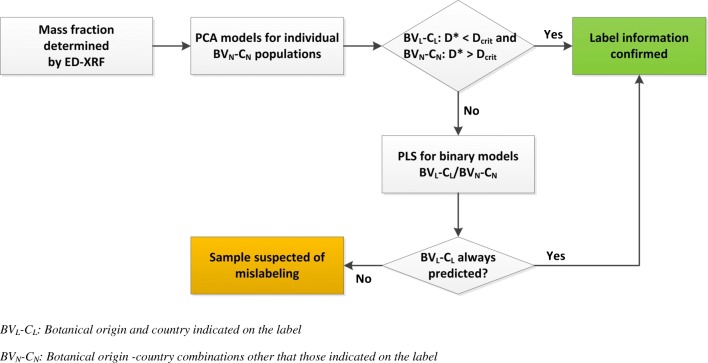
Flow chart from analysis to evaluation of label information

The approach used is summarised in Fig. 4: each of the honeys used for external validation was tested against each of the available PCA models. When a honey sample was not flagged as an outlier by the model of a BV-C combination different from the one indicated on the label, the PLS-DA model created for that BV-C combination and the one indicated on the label (BV_L_-C_L_), was used for classification purposes.

For some botanical varieties such as thyme, sunflower and most dark honeys (chestnut, eucalyptus, lime, manuka, heather), the number of honeys available was low and the pre-requisite of five honeys available per BV-C group was only fulfilled in some cases. In the particular case of dark honeys, five samples were only available for the groups chestnut-Italy, lime-Romania and manuka-New Zealand. For this reason, PLS-DA models were created not only for populations with five or more samples but also for classes for which three samples were available: chestnut-Spain, eucalyptus-Spain, thyme-Spain, thyme-New Zealand, robinia-Romania and sunflower-Romania. Those models were also validated externally with honey samples of a different production year and brand than those used in the construction of the models.

Out of the 11 samples used for external validation, ten were properly classified using PLS-DA models. Robinia-Romania was wrongly classified as robinia-Hungary unless five principal components were used to construct the PLS-DA model. It needs to be kept in mind that only three robinia-Romania samples were available for the construction of the model while, with 11 samples, robinia-Hungary was one of the groups better represented in our repository.

Predictions were also successful when using PLS-DA models constructed for populations in which only three samples were available. For instance, one chestnut-Spain honey was correctly classified as Spanish honey by a PLS-DA model created for Italian (*n* = 5) and Spanish (*n* = 3) chestnut honeys (Fig. S2 in the ESM) and as chestnut honey by a PLS-DA model constructed with chestnut-Spain (*n* = 3) and eucalyptus-Spain (*n* = 3) honeys. One lime-Romania honey was also correctly classified when compared to other dark honeys from different geographical origin.

The good performance of the PLS-DA models demonstrates that profiling by ED-XRF is an appropriate tool for verifying botanical and geographical origin claims. The mass fractions obtained in this work are in good agreement with those previously reported in the literature [9, 11, 12] using more sophisticated techniques such as ICP-AES and ICP-MS. The method used in this work did not require any sample treatment, which increases the sample throughput and reduces the environmental impact of the analysis, since no harsh reagents need to be used.

Although the list of elements quantifiable in honey by ICP-based techniques is larger than by ED-XRF due to the lower LoQs that characterise the former, only a reduced number of elements is of real relevance for modelling and classification purposes, i.e. Ba, Ca, Fe, Na, Sn, Al, B, Cu, K, Mg, Mn, Ni, P, Rb, Sr and Zn according to Zhou et al. [3]; Ca, K, Mg, Na, P and S according to Czipa et al. [9]; and Ca, K, Mg, Li, Na and Rb for botanical variety and Fe, Zn, Cu, Cr, Ni, Al, Cd and Pb for geographical origin according to Batista et al. [12] which can even be reduced further to Mg, Na and K to improve the prediction capacity although not in all types of honey.

Determination of halogens such as Cl and Br by ED-XRF is straightforward while careful optimisation of the digestion procedure is fundamental when ICP-based methods are used [17]. For all the BV-C populations, Cl contributed strongly to the definition of the model.

ED-XRF when compared to ICP-based techniques is characterised by higher LoQs and for this reason only Al, P, Cl, K, Ca, Fe, Zn, Mn, Rb and Br could be determined in most honeys, although not in all. Other elements that could be randomly present in some honeys in small quantities, for instance due to contamination during processing and bottling or to the use of specific fertilisers in some fields, were not detected. The mass fractions of those elements would have diluted the important information increasing the background noise of the data used for modelling. The amount of quantifiable elements could be increase with a careful optimisation of the ED-XRF method and the calibration curve used.

The correct classifications rate achieved in this work is comparable to other previously published in the literature ranging from 90 to 100% [3, 9–12]. It needs to be emphasised that in this work, the classification capacity of the models was validated via external validation with samples from different brands and production year than those used to construct the classification models. Most other works used either cross-validation or splitting of the available samples in two sets, one to build up the models and the second one for validation purposes, using only honey samples perfectly characterised for geographical origin and/or botanical variety without including unknown samples in the validation. Also some of those works use next to the elemental mass fractions some other parameters for classification purposes, i.e. stable isotopic ratios [3, 10], water, total protein content, electric conductivity, pH, specific rotation, colour, free acids, lactones [10], ash percentage, insoluble matter, reducing sugars and diastase activity [11].

The approach followed in this work seems to be quite robust achieving a satisfactory performance with a reduced number of variables and observations in some models. Few samples are enough to construct models for classification purposes if the samples are chosen in such a way that they represent the maximum variability that characterises a certain population.

## Conclusions

Profiling of the elemental composition of honeys by ED-XRF proved to be an appropriate technique for confirming the botanical variety and geographical origin of the honeys as indicated on the labels under which they are commercialised. ED-XRF is a high-throughput multielemental analytical technique that does not require complicated sample pre-treatment, thus being ideal for routine control analyses.

The built-in calibration curves provided by the manufacturer of the ED-XRF instrument have allowed the successful classification of a wide variety of European honeys. A careful optimisation of the method, for instance of the irradiation time and of the amount of honey used for the analysis, could increase the sensitivity of the method and therefore also the number of elements to be used as variables in the multivariate analysis, which would increase the prediction capability of the models.

Control laboratories should focus on the construction of models that cover the botanical varieties and geographical origins most relevant for their routine work. The predictive capabilities of this chemometric approach will benefit an initial effort to collect a representative number of authentic samples whose botanical and geographical origin is sufficiently documented.

## Electronic supplementary material

ESM 1 (PDF 74.5 kb).
