# Supplementary material for: Use of energy-dispersive X-ray fluorescence combined with chemometric modelling to classify honey according to botanical variety and geographical origin
Source: Anal Bioanal Chem. 2019 Nov 25;412(2):463–72. doi: 10.1007/s00216-019-02255-6 (PMC6992546; doi:10.1007/s00216-019-02255-6)
Supplement: Supplementary file 1 — (PDF 74.5 kb). [file 216_2019_2255_MOESM1_ESM.pdf]

Analytical and Bioanalytical Chemistry

Electronic Supplementary Material

Use of energy-dispersive X-ray fluorescence combined with chemometric modelling to classify honey according to botanical variety and geographical origin

Yiannis Fiamegos, Catalina Dumitrascu, Michele Ghidotti, Maria Beatriz de la Calle Guntiñas

a)

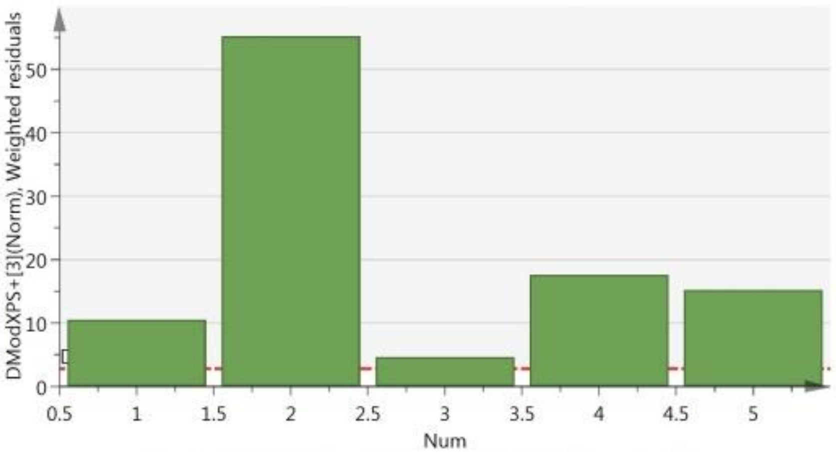

b)

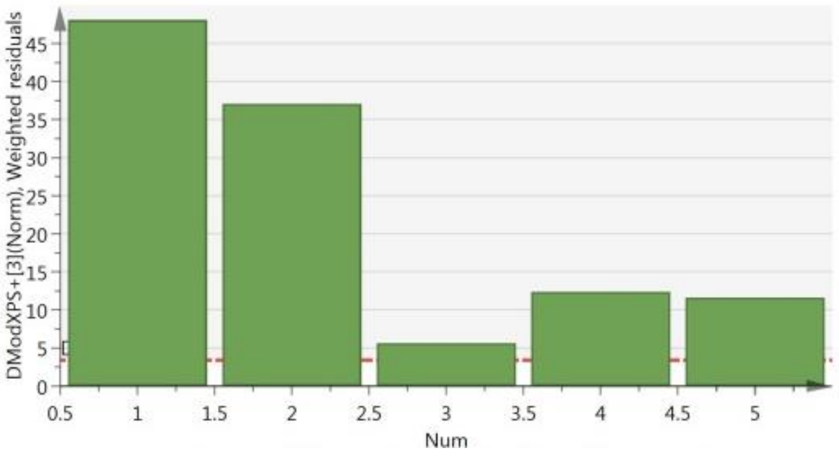

Fig. S1 Y-axis: Mahalanobis distance, DModX PS+ (Dotted red line: Critical Mahalanobis distance, D-crit, at 95 % CI), of five Spanish lavender honeys to the model of: a) Orange-Spain, and b) Lavender- Portugal [16], X-axis: individual sample number

a)

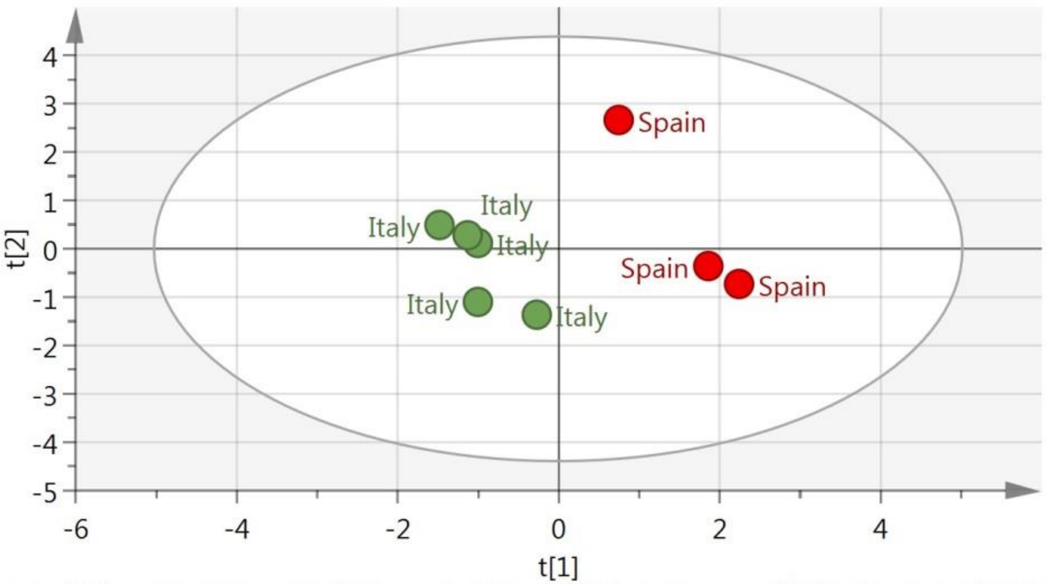

Fig. S2 PLS model created for chestnut-Italy and chestnut-Spain. t[1] and t[2] refer to 1st and 2nd principal components respectively
